# Beyond Access to Sanitation Services: How Maternal Education Moderates Childhood Diarrhea Risk in Indonesia’s Multilevel Context

**DOI:** 10.34172/jrhs.11228

**Published:** 2025-10-18

**Authors:** Ika Dharmayanti, Dwi Hapsari Tjandrarini, Rina Marina, Khadijah Azhar, Basuki Rachmat, Zahra Zahra, Tities Puspita, Sri Irianti, Doni Lasut, Andre Yunianto

**Affiliations:** ^1^Research Center for Public Health and Nutrition, National Research and Innovation Agency, Cibinong, West Java, Indonesia; ^2^Health Development Policy Agency, Ministry of Health, Jakarta, Indonesia; ^3^Research Centre for Limnology and Water Resources, National Research and Innovation Agency, Cibinong, West Java, Indonesia

**Keywords:** Diarrhea, Sanitation, Maternal education, Poverty, Health equity

## Abstract

**Background:**

Despite long-standing efforts to improve water, sanitation, and hygiene (WASH) infrastructure in Indonesia, childhood diarrhea remains a pressing public health concern. This study focuses on the gaps between infrastructure and health equity by examining the intertwined effects of child, household, and environmental factors on the risk of diarrhea.

**Study Design:**

A cross-sectional study.

**Methods:**

In this study, data from the 2017 Indonesia Demographic and Health Survey (IDHS) were analysed using a multilevel logistic regression model. The survey included 16632 children, with children nested within households and households within a cluster (environment). All child, household, and environmental-level variables were included as fixed effects. Cross-level interactions were examined with sanitation, maternal education, and household wealth in terms of the prevalence of diarrhea.

**Results:**

Children aged 12–23 months (AOR=4.24; 95% CI: 3.23, 5.43), those with low birth weight [AOR=1.33 (95% CI: 1.04-1.70)], and those born to mothers with low education (AOR=1.74; 95% CI: 1.25, 2.44) had significantly higher odds of experiencing diarrhea. A significant interaction revealed that the impact of poor sanitation on children with less-educated mothers (AOR=1.68; 95% CI: 1.19, 2.37) and among educated mothers in children from low-income households (AOR=1.6; 95% CI: 1.12, 2.29) remained elevated. Children in non-Java-Bali regions also had persistently higher rates of diarrhea.

**Conclusion:**

Access to sanitation is insufficient to guarantee health equity. Maternal education plays a crucial moderating role in translating infrastructure into better health outcomes. To accomplish Sustainable Development Goals 6 and 10, integrated equity-focused sanitation programs, including poverty reduction and maternal empowerment, are of great importance.

## Background

 Diarrhea remains a serious health concern, causing morbidity and mortality in children under five globally. There are around 1.7 billion cases and 443­832 deaths per year in low- and middle-income countries (LMICS) due to inadequate water, sanitation, and hygiene (WASH) and healthcare facilities.^1^ Diarrhea affects 12.3% of Indonesian children, according to the Ministry of Health, with higher rates observed in rural areas and undeveloped provinces.^2^ The prevalence of diarrhea-associated deaths in newborns and infants ranged from 5% to 7%,^3,4^ highlighting major problems in access to healthcare and infrastructure.

 Clinical definitions emphasize pathogen transmission through contaminated water or inadequate hygiene.^5^ Infrastructure-based interventions in water, sanitation, and hygiene (WASH) programs face limited efficacy. Studies in Bangladesh and Kenya have shown that access to improved WASH services has a limited effect on children’s linear development and incidence of diarrhea,^6,7^ implying that behavioral and socioeconomic factors at the household level are crucial. Maternal education has a significant impact on health outcomes, as educated mothers are more likely to maintain proper hygiene, identify symptoms early, and seek medical care.^8,9^ However, maternal education accounts for only 15% of the decrease in infant mortality between 1980 and 2015 in Indonesia,^10^ underscoring the importance of intersecting disparities. Households experiencing poverty encounter multiple overlapping risks, including overcrowded living conditions, limited access to healthcare, and inconsistent WASH services, all of which exacerbate environmental health challenges.^11^

 This interaction between maternal, infrastructure, and socioeconomic disparity reveals a structural gap in how WASH policy is implemented. While Indonesia has made progress in expanding its infrastructure, the ongoing prevalence of diarrhea suggests that its benefits are not being shared fairly. Previous research has demonstrated that the educational and economic background of the household may influence consumption, maintenance, and supplementary hygiene habits, even when improved water or toilets are provided.^12^

 Regional differences further complicate the disease incidence in Indonesia. The eastern provinces are suffering from disproportionately high rates of under-five mortality, diarrhea, and stunting, rather than Java-Bali.^13,14^ However, few studies have systematically examined the cross-regional connections between structural and maternal-level factors. Most previous research employed single-level analyses, which overlooked the nested structure of child health determinants, including children within families and households within clusters, as well as their interactions. A study shows that upgraded toilets reduce the risk of diarrhea by 22%, but this impact is reduced by 71% in poor households due to maintenance problems.^12^ These findings highlight the importance of investigating the relationships between socioeconomic position, infrastructure condition, and parenting practices at multiple levels.

 Using the Indonesia Demographic and Health Survey (IDHS), we developed a multilevel analysis to evaluate direct and interaction effects. This approach provides a comprehensive assessment of risk factors for diarrhea, overcoming the limitations of research that examines WASH conditions independently. Based on the Developmental Origins of Health and Disease (DOHaD) concepts, our study examines how early-life exposures, such as inadequate sanitation, maternal disadvantage, and poor drinking water quality, influence health outcomes in children under five.^15,16^ Our study enhances the understanding of intergenerational health disparities in Indonesia by examining the combined effects of maternal education, household poverty, and access to WASH.

## Materials and Methods

###  Data Source 

 This study utilized data from the 2017 IDHS. This nationally representative cross-sectional survey was carried out by Statistics Indonesia in collaboration with the National Population and Family Planning Board and the Ministry of Health.^17^ The survey employed a stratified multistage sample that covered 1970 census blocks and 49250 households, with a household response rate of 99.5%. The women’s module received replies from 49627 women between the ages of 15 and 49 (97.8%). The current study focused on 16632 children under the age of five from eligible households.

###  Study Variables

 The outcome variable was the incidence of diarrhea among children under five, as reported in question 608 of Section 6: Child Health and Nutrition in the 2017 IDHS. Diarrhea status was determined as a binary outcome (1 = Yes, 0 = No) based on maternal reports of whether the child had diarrhea in the two weeks preceding the survey. This approach adheres to the standard definition and measurement protocol used in Demographic and Health Surveys (DHS), which are widely utilized in child health research.^18^

 Child-level factors included age (in months), the child’s gender, and birth weight ( ≥ 2500 g or < 2500 g). Maternal education was classified into three categories: no education/primary, secondary, and higher education. Maternal age was also considered. The household wealth index, created by DHS, was divided into quintiles (poorest to richest). Access to improved water, sanitation, and hygiene, as determined by the WHO/UNICEF Joint Monitoring Program (JMP), is classified as improved or unimproved.^19^ Residence type (urban-rural) and geographical region are contextual factors that capture unobserved regional heterogeneity influencing child health. For interaction analysis, low maternal education was defined as lacking formal education, while educated mothers had at least a secondary education.

 A multilevel analytical approach was used to account for the hierarchical data structure, with children nested within households and households within clusters. Figure S1 (Supplementary file 1) presents the conceptual framework, showing how child-, maternal/household-, and environmental factors influence the risk of childhood diarrhea. The framework reflects both the theoretical pathways that impact child health and the hierarchical data structure of the multilevel model, which nests children within families, families within households, and households within clusters. Environmental variables, such as geography and residence type, were treated as fixed effects to allow for greater contextual control.

###  Statistical Analysis

 Descriptive statistics were used to summarize all study variables, while bivariate analysis examined their association with diarrhea status among children under five. A multilevel logistic regression model was used to account for the hierarchical structure of the data, with children (Level 1) nested within households (Level 2), which in turn are nested within clusters or primary sampling units (PSUs) at Level 3. A multistage stratified sampling method was used in this study and potential intraclass correlation and non-independence of observations within higher-level units were examined. Multilevel modeling is suggested for evaluating DHS data because it improves standard error estimation, compensates for clustering effects, and allows for the analysis of both individual and contextual level health outcomes.^20-22^

 The modeling process involved several key assumptions. First, the nested data structure justified the use of a multilevel logistic regression approach to account for clustering and the non-independence of observations across levels.^23,24^ Second, it is assumed that Level 1 outcomes are independent, given the fixed and random effects. Including random effects accounts for within-household and within-cluster correlations. Third, multicollinearity was assessed using the variance inflation factor (VIF). All VIF values were below 2, indicating no serious multicollinearity issues.^25^ Fourth, predictor variables were selected based on theoretical frameworks and previous literature. All relevant variables were kept constant across models to avoid omitted variable bias. We evaluated model fit using Akaike’s information criterion (AIC), the Bayesian information criterion (BIC), and likelihood ratio tests.^26^ These criteria were used to select the best models based on the balance between fit and parsimony.

 Clusters were defined as the PSUs of the survey. To account for unobserved heterogeneity and intra-cluster similarity, that is, the tendency of individuals within the same group to have more similar outcomes than those in different groups, random intercepts were specified at both the household (Level 2) and cluster (Level 3) levels. The random effects were assumed to adhere to a normal distribution (mean = 0, variance estimated). The appropriateness of the multilevel modeling approach was evaluated, and the degree of clustering was quantified by calculating the intraclass correlation coefficient (ICC) and the median odds ratio (MOR). These measures are designed to capture the proportion of outcome variance attributable to higher-level units and to indicate the extent to which contextual factors at the household or cluster level influence individual-level outcomes.

 We employed a sequential model-building strategy to assess the impact of incorporating higher-level variables on the outcome and the significance of the predictors.^23^ Model 0 (Null Model) was a baseline model that included only random intercepts at the household and cluster levels, with no covariates. It was used to estimate ICCs. Model 1 included child-level covariates, such as age, gender, and birth weight. Model 2 extended Model 1 by adding household-level variables, including maternal age, maternal education, household wealth index, and WASH indicators. Model 3 added cluster-level contextual variables to Model 2, specifically the type of residence and region. Model 4 (Full Model) included all the variables from the previous models, as well as selected cross-level interaction terms based on their theoretical relevance.

 This step-by-step technique employed a hierarchical conceptual framework, progressing from individual to household and cluster levels. This method enables the identification of confounding or mediating effects across levels. All theoretically essential variables were included in each model, regardless of statistical significance, to ensure consistency and reduce omitted variable bias.

 Throughout this process, all theoretically relevant variables were included in each model to ensure comparability and minimize the risk of omitted variable bias. Changes in the magnitude or significance of the coefficients across the models were used to infer potential confounding factors, shared variance, or effect modification. This approach enabled us to evaluate how associations between predictors and childhood diarrhea outcomes changed at different levels of influence as outlined by Snijders and Bosker.^23^

 To account for the complex survey design, the analysis incorporated sampling weights, strata, and PSUs. Model comparison was guided by the AIC, wherein models with ΔAIC < 2 were considered equally credible.^26^ As a result, while numerous intermediate models were tested, only three models (the null model, an intermediate model incorporating child and household predictors, and the final model) were reported in the results for clarity and interpretability. Statistical significance was established at a *P*-value threshold of < 0.05. Adjusted odds ratios (aORs) were used to report the final results with 95% confidence intervals (CIs). All statistical analyses were carried out using Stata version 17.

 To evaluate the robustness of our findings, we conducted a sensitivity analysis on a randomly selected 30% subset of the original dataset (N = 4990). We applied the same modeling framework, including the survey design specifications and model selection criteria, to this reduced sample. The goal of this analysis was to determine if the observed associations remained consistent when using a substantially smaller yet representative subset of the data.^27^

## Results

###  Sample Characteristics

 Our analysis included 16632 children under the age of five. The incidence of diarrhea in the two weeks before the survey was 14.2% (n = 2370). Interestingly, the prevalence of diarrhea was similar across all age groups of children, with the highest frequency observed among children aged 12-23 months (19.9%) and the lowest among those aged 48-59 months (9%).

Table 1 presents the distribution of respondents based on child characteristics, household and maternal factors, and environmental factors. Bivariate analysis revealed a significant relationship between the prevalence of diarrhea and child age, maternal, household, and environmental factors, except for access to hygiene facilities. The multilevel models then included these variables to evaluate the adjusted relationships while accounting for clustering effects.

**Table 1:** Frequency distribution of respondents by different characteristics in Indonesia

**Variables**	**Overall sample, n=16632**		**Diarrhea, n=2370**		**Crude OR (95% CI)**	***P *** **value**
	**Number**	**Percent**	**Number**	**Percent**		
Child gender						
Female	8091	49.1	1077	13.7	Ref.	
Male	8541	50.9	1293	14.6	1.08 (0.97, 1.20)	0.151
Child age (months)						
48–59	3384	20.3	304	9.0	Ref	
36–47	3309	20.0	401	12.1	1.38 (1.13, 1.69)	0.001
24–35	3293	19.8	545	15.8	1.90 (1.59, 2.26)	0.000
12–23	3412	20.6	696	19.9	2.51 (2.11, 3.00)	0.000
0–11	3234	19.3	424	13.9	1.63 (1.35, 1.97)	0.000
Birth weight (g)						
≥ 2,500	15366	93.1	2156	14.0	Ref	
< 2,500	1266	6.9	214	16.8	1.25 (1.01, 1.54)	0.038
Maternal age (year)						
35–49	4997	29.5	625	12.4	Ref	
25–34	8683	52.1	1184	13.5	1.10 (0.97, 1.25)	0.138
15–24	2952	18.4	561	18.9	1.65 (1.41, 1.91)	0.000
Maternal education						
Higher	2951	15.2	331	10.5	Ref	
Secondary	9301	57.9	1343	14.3	1.42 (1.20, 1.68)	0.000
Primary/no education	4380	26.9	696	15.9	1.61 (1.34, 1.93)	0.000
Household wealth index						
Richest	2811	19.0	279	10.2	Ref	
Richer	2938	20.3	407	14.4	1.49 (1.21, 1.82)	0.000
Middle	3077	20.4	444	14.2	1.46 (1.19, 1.78)	0.000
Poorer	3253	20.1	505	15.8	1.66 (1.37, 2.01)	0.000
Poorest	4553	20.2	735	16.0	1.67 (1.38, 2.04)	0.000
Drinking water						
Improved	14721	90.4	2046	13.8	Ref	
Unimproved	1911	9.6	324	17.8	1.35 (1.13, 1.60)	0.001
Toilet facilities						
Improved	13456	81.4	1809	13.4	Ref	
Unimproved	3176	18.6	561	17.8	1.40 (1.23, 1.60)	0.000
Hygiene facilities						
Improved	7902	48.1	1107	14.1	Ref	
Unimproved	8730	51.9	1263	14.2	1.01 (0.90, 1.13)	0.923
Residential area						
Urban	8169	48.5	1071	13.0	Ref	
Rural	8463	51.5	1299	15.3	1.21 (1.07, 1.38)	0.003
Region						
Java-Bali	5087	55.6	648	13.0	Ref	
Non-Java-Bali	11545	44.4	1722	15.6	1.23 (1.09, 1.40)	0.001

**Figure 1:**
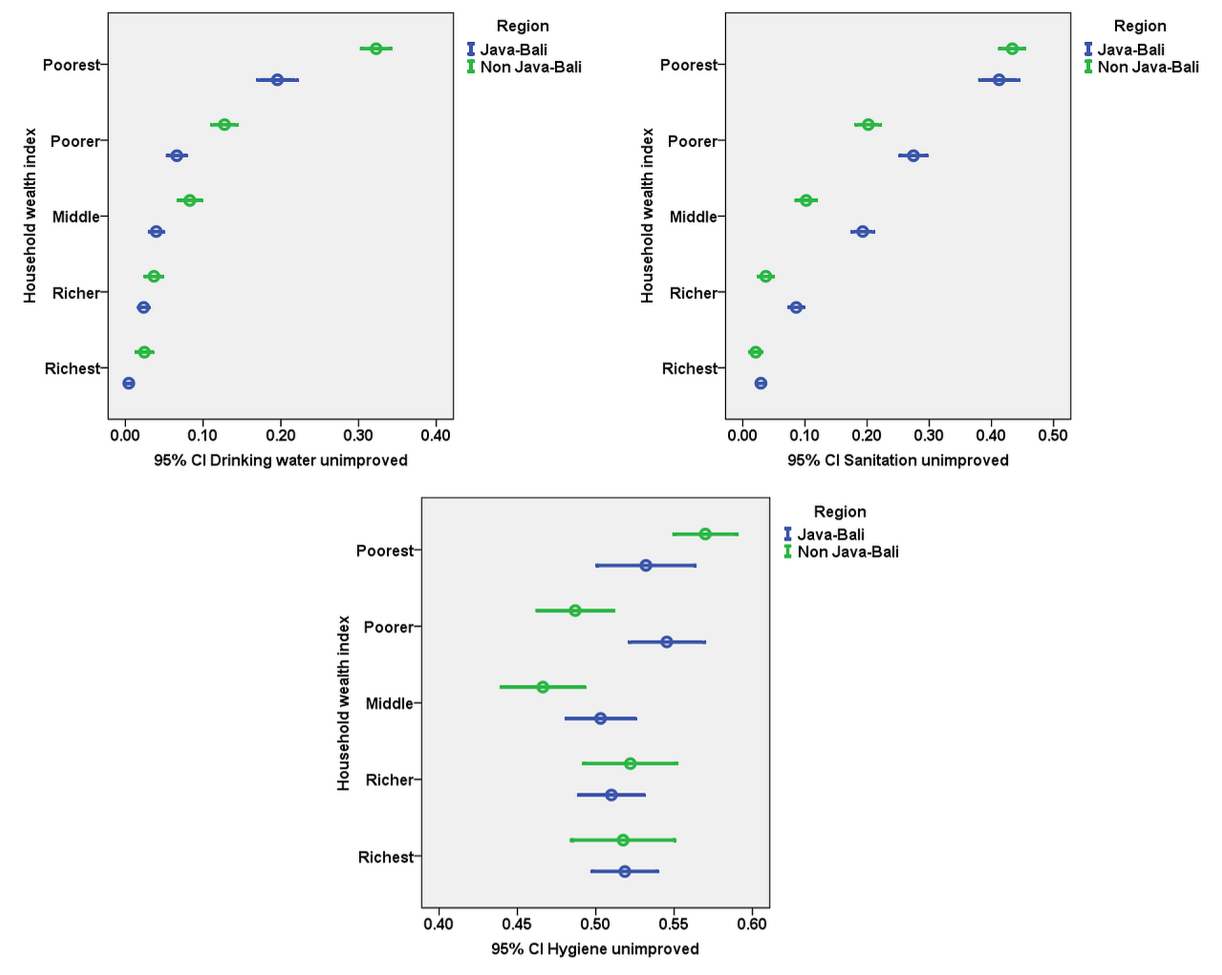
Disparities in access to WASH services by household wealth and region

Figure 1 shows the percentage of families having unequal access to drinking water, sanitation, and hygiene by region (Java-Bali vs. non-Java-Bali) and across wealth quintiles. Regarding access to drinking water and sanitation, improvements are observed with increasing wealth, while the Non-Java-Bali region consistently exhibits inferior conditions, particularly for the poorest population. Conversely, unimproved hygiene behaviors are not influenced by location or socioeconomic status, as they are prevalent across all wealth levels and exhibit minimal regional variation. Promoting hygiene requires a universal, behavior-based strategy, even though water and sanitation initiatives should focus on the most impoverished and remote areas.

###  Multilevel Model 

Table 2 presents the results of the multilevel logistic regression model, showing factors significantly associated with childhood diarrhea. The final model identified the critical factors associated with childhood diarrhea. Compared to children aged 48-59 months, those aged 12-23 months had the highest odds of diarrhea [aOR = 4.24 (95% CI: 3.23-5.43)]. Children with low birth weight ( < 2500 g) had higher odds of diarrhea [aOR = 1.33 (95% CI: 1.04–1.70)] compared to those with normal birth weight ( ≥ 2,500 g). Children of mothers aged 15–24 years had higher odds of diarrhea than those of mothers aged 35–49 years [aOR = 1.77 (95% CI: 1.42–2.19)]. Regional differences were also evident; in other words, living in non-Java-Bali regions was linked with higher odds of diarrhea compared to living in Java-Bali regions [aOR = 1.26 (95% CI: 1.06-1.52)].

**Table 2:** Factors associated with diarrhea among children under five

**Variables (n=16632)**	**Model 2 (household) **		**Model 4 (with interaction) **	
	**OR (95%CI)**	***P *** **value**	**OR (95%CI)**	***P *** **value**
Child’s age (months)				
48–59	Ref.		Ref.	
36–47	1.63 (1.28, 2.06)	0.000	1.62 (1.28, 2.04)	0.000
24–35	2.84 (2.23, 3.62)	0.000	2.80 (2.21, 3.54)	0.000
12–23	4.37 (3.39, 5.63)	0.000	4.24 (3.32, 5.43)	0.000
0–11	1.88 (1.48, 2.38)	0.000	1.86 (1.47-2.35)	0.000
Child’s gender				
Female	Ref.		Ref.	
Male	1.32 (1.15, 1.51)	0.000	1.31 (1.14, 1.50)	0.000
Child’s birth weight (g)				
≥ 2,500	Ref.		Ref.	
< 2,500	1.35 (1.05, 1.74)	0.020	1.33 (1.04, 1.70)	0.023
Mother’s age (year)				
35–49	Ref.		Ref.	
25–34	1.15 (0.97, 1.37)	0.099	1.15 (0.97, 1.35)	0.100
15–24	1.79 (1.44, 2.24)	0.000	1.77 (1.42, 2.19)	0.000
Mother’s educational level				
Higher	Ref.		Ref.	
Secondary	1.21 (0.97, 1.51)	0.091	1.25 (1.00, 1.55)	0.046
No formal education/primary	1.42 (1.10, 1.84)	0.008	1.74 (1.25, 2.44)	0.001
Household wealth				
Richest	Ref.		Ref.	
Richer	1.53 (1.17, 1.99)	0.002	1.45 (1.12, 1.89)	0.005
Middle	1.58 (1.21, 2.08)	0.001	1.43 (1.09, 1.87)	0.010
Poorer	1.67 (1.27, 2.19)	0.000	1.03 (0.69, 1.55)	0.885
Poorest	1.62 (1.22, 2.16)	0.001	0.98 (0.65, 1.47)	0.923
Drinking water				
Improved	Ref.		Ref.	
Unimproved	1.11 (0.87, 1.40)	0.400	1.07 (0.85, 1.35)	0.567
Toilet facilities				
Improved	Ref.		Ref.	
Unimproved	1.29 (1.01, 1.66)	0.045	1.06 (0.79, 1.40)	0.718
Hygiene facilities				
Improved	Ref.		Ref.	
Unimproved	0.98 (0.85, 1.14)	0.836	0.99 (0.86, 1.14)	0.856
Residential area				
Urban	-	-	Ref.	
Rural	-	-	1.05 (0.88, 1.26)	0.556
Region	-	-		
Java-Bali	-	-	Ref.	
Non-Java-Bali	-	-	1.26 (1.06, 1.52)	0.011
Interaction terms				
Unimproved toilet × low-educated mother	-	-	1.68 (1.19, 2.37)	0.003
Low household wealth × educated mother	-	-	1.60 (1.12, 2.29)	0.010
Akaike information criterion (AIC)	13091.41	-	13083.17	-
Bayesian information criterion (BIC)	13245.79	-	13268.42	-
Intraclass correlation coefficient (ICC)	0.55	-	0.59	-
Median odds ratio (MOR)	7.78	-	7.90	-
ICC (cluster)	0.06	-	0.06	-
MOR (cluster)	1.96	-	1.96	-

 In Model 2, a lower maternal education level was significantly associated with an increased odds of childhood diarrhea. Compared to mothers with a higher level of education, mothers with no formal education or only primary education had 1.42 times higher odds [aOR = 1.42 (95% CI: 1.10–1.84)]. Similarly, household wealth was strongly inversely associated with diarrhea; in other words, the poorest households had significantly higher odds than the wealthiest households [aOR = 1.62 (95% CI: 1.22–2.16)].

 However, in the final model (Model 4), which included interaction terms and contextual variables, the main effects of household wealth and sanitation lost statistical significance. The odds of diarrhea among children in the poorest group were significantly higher in Model 2 (*P* = 0.001) but were no longer significant in Model 4 (*P* = 0.923). Similarly, the effect of unimproved sanitation was significant in Model 2 (*P* = 0.045) but became non-significant in the final model (*P* = 0.718).

 These changes reflect significant interaction effects. Specifically, including unimproved toilet facilities and low maternal education was significantly associated with an increased likelihood of diarrhea [aOR = 1.68 (95% CI: 1.19–2.37)], as was the interaction between low household wealth and an educated mother [aOR = 1.6 (95% CI: 1.12–2.52)]. The results indicate that the protective effect of maternal education may be limited in conditions of material deprivation. Similarly, the adverse impact of unimproved sanitation is amplified when combined with low maternal education, indicating compounded vulnerability. These findings underscore the importance of addressing both academic and structural inequities simultaneously to reduce the risk of childhood diarrhea.

**Figure 2:**
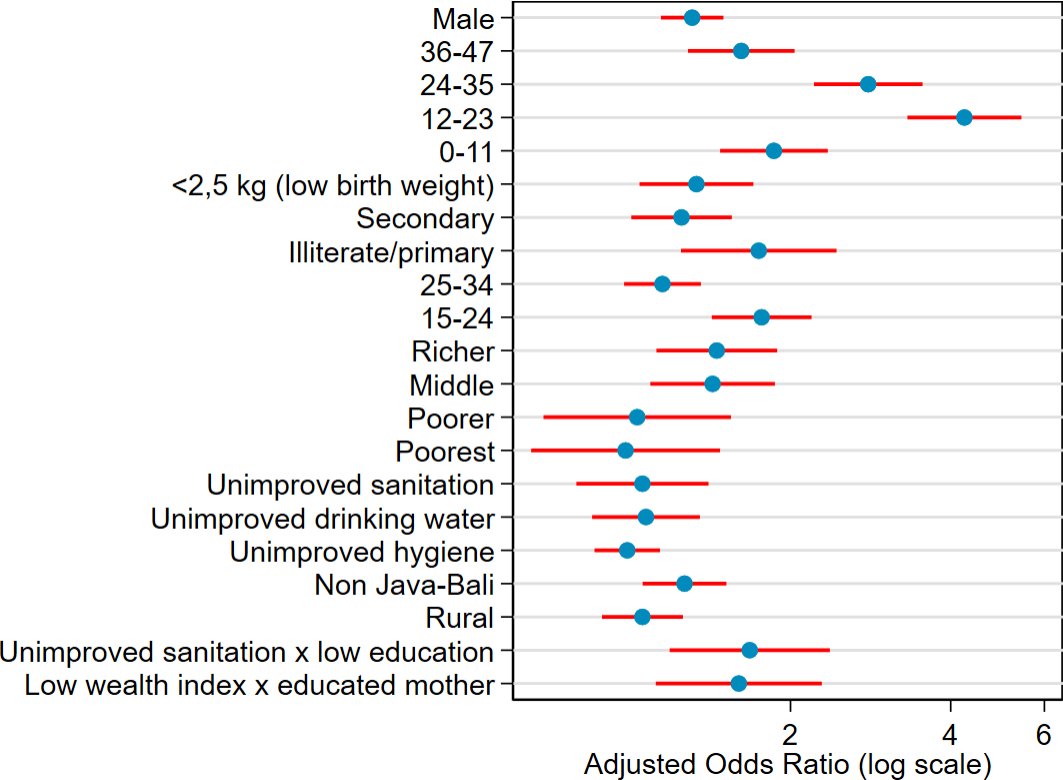
A forest plot of the adjusted odds ratios (aORs) based on the multilevel model

 These adjusted odds ratios from the multilevel final model are visualized in Figure 2. Child age (especially 12-23 months), low birth weight, maternal age (15-24 years), low maternal education, and family household index are all significant predictors of diarrhea in children. The most enormous impact was seen in children aged 12 to 23 months, with an aOR greater than 4.0. The interaction of poor toilet facilities and poor maternal education, as well as low-income families with educated mothers, highlights the impact of overlapping structural disadvantages. It illustrates how personal and environmental factors influence the likelihood of diarrhea.

**Figure 3:**
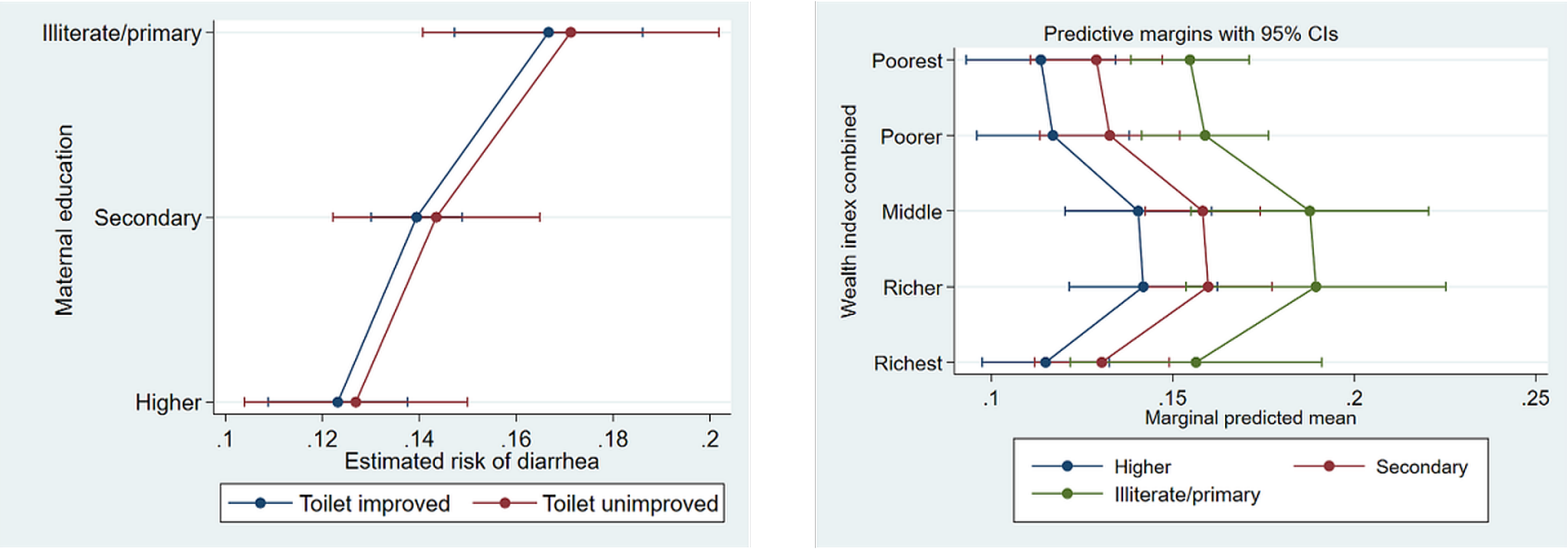
The effect of the interaction between maternal education and sanitation facility and wealth on the predicted probability of childhood diarrhea

 Margin plots (Figure 3) further illustrate the interaction patterns, showing that maternal education moderates the risk of childhood diarrhea by interacting with sanitation (Panel a) and wealth (Panel b). These plots highlight that the highest risk of diarrhea is among children with low-educated mothers and unimproved sanitation or low household wealth. Although the interaction with sanitation is statistically significant, its effect appears localized and may vary across subgroups, which suggests that the moderating role of education is complex.

 The null model (Model 0) revealed significant variance in diarrhea risk at both the household (ICC = 0.476; MOR = 5.92) and cluster levels (ICC = 0.073; MOR = 2.01), supporting the use of multilevel modeling. In Model 2, the household ICC increased to 0.550. In contrast, the cluster ICC decreased to 0.059, implying that individual- and household-level variables explained some between-cluster variations but revealed persisting heterogeneity within households (MOR = 7.78). The final model (Model 4), which included all main effects and significant interactions, produced an increased ICC (0.588 households; 0.062 clusters) and a high household-level MOR (7.90), indicating that key predictors reduced some variance but left significant unexplained heterogeneity, particularly at the household level. This highlights the need for both household-level interventions and broader structural reforms.

 A sensitivity analysis yielded patterns consistent with the main findings (Supplementary file 1, Table S1). Reductions in the MOR (7.90 to 6.82) and the ICC (0.588 to 0.552) were observed at the household level, suggesting a potential decrease in statistical power. Conversely, cluster-level variation increased slightly (MOR: 1.96 to 2.17; ICC: 0.062 to 0.090), indicating stable and robust between-cluster effects despite smaller sample sizes.

## Discussion

 This study offers a new perspective on the complex and overlapping factors contributing to diarrhea among children in Indonesia by examining how maternal education, family poverty, and access to WASH facilities, particularly sanitation, interact to influence health outcomes. The findings underscore that individual or household-level behaviors do not solely drive diarrhea but are embedded within broader socioeconomic and systemic disparities.

 Children aged 12 to 23 months exhibited the highest risk of diarrhea, aligning with prior research identifying this developmental window as particularly vulnerable due to declining maternal immunity, dietary transitions during weaning, and increased environmental exploration.^28,29^ Ningsi et al noted that mobility at this age significantly heightens exposure to contaminated surfaces.^30^

 In line with earlier studies, low birth weight emerged as a significant risk factor, likely reflecting intergenerational effects of maternal malnutrition and limited antenatal care, which compromise neonatal immunity.^31^ Preventive strategies such as maternal nutrition support, improved antenatal services, and promotion of exclusive breastfeeding can reduce susceptibility of infants to enteric infections.^32^

 Young maternal age (15–24 years) was also associated with an increased prevalence of diarrhea, echoing findings from Indonesia and Sub-Saharan Africa.^33,34^ Younger mothers often face intersecting barriers, including lower health literacy, economic vulnerability, and limited decision-making power, which hinder their ability to provide adequate childcare.^30^ These results support the need for targeted interventions for adolescent and young mothers, including education, mentoring, and economic support programs.

 Socioeconomic status remained a strong predictor of diarrhea. Children from poorer households and those with mothers who are less educated consistently showed higher odds of diarrhea, which aligns with the Social Determinants of Health (SDH) framework.^35,36^ Poverty influences access to safe water, adequate sanitation, nutritious food, and healthcare, while maternal education shapes knowledge, behaviors, and health-seeking practices.^11,37^

 Unimproved sanitation did not significantly increase the risk of diarrhea, reinforcing the fecal-oral transmission route, especially in contexts of open defecation or shared facilities.^29,38^ Although this study found no significant correlation between better water availability and diarrhea, this conclusion is consistent with other research showing that access is insufficient if water is not treated or appropriately stored.^39,40^ Therefore, community water governance systems and a shift in hygiene behavior must be combined with infrastructure expenditures.

 Although our adjusted models did not reveal a statistically significant link between household-level hygiene facilities and childhood diarrhea, this finding aligns with recent research. It suggests that simply providing hygiene infrastructure may be insufficient to drive effective hygiene behaviors without the support of education and behavior change interventions.^40,41^ WHO/UNICEF reveal that while handwashing facilities are frequently provided, they are rarely used or lack key components such as soap or running water.^42^ Additionally, our binary measure of hygiene access may not fully capture the nuances of real-world hygiene practices, which are heavily influenced by behavioral, cultural, and societal norms.

 The results revealed notable regional inequalities, with children living outside Java-Bali experiencing significantly higher incidence rates of diarrhea, even after adjusting for household characteristics. These findings highlight long-standing governance and infrastructural inadequacies in eastern Indonesia and other distant locations.^43^ Ignoring these spatial inequities may result in chronic health disparities despite national gains in infrastructure development.

 Our findings align with the DOHaD framework, which posits that early-life exposures, particularly during the first 1000 days of life, have lasting effects on physical, cognitive, and metabolic health.^16,44^ Repeated diarrhea episodes in early childhood have been linked to stunting, impaired brain development, and increased risk of adult non-communicable diseases, reinforcing the urgency of early WASH and maternal health interventions.

 This study also reinforces the capability approach to equity in WASH services. While access to infrastructure is necessary, it is insufficient without the knowledge, autonomy, and contextual resources to use facilities effectively.^45,46^ Our study demonstrates that the protective effect of maternal education on childhood diarrhea is modulated by household income and sanitation. Even children of educated mothers living in low-income households were at high risk, consistent with studies in LMICs showing that maternal literacy alone cannot reduce diarrhea without accompanying economic changes.^47,48^

 Furthermore, low-income and low-education settings frequently hinder the practical application of health knowledge; in contrast, educated mothers in better-resourced households can more effectively apply hygiene and sanitation behaviors. Similarly, the interaction between children with unimproved sanitation was especially vulnerable when their mothers had lower levels of education. The findings are consistent with evidence that combined WASH and maternal health programs emphasize the need for a multidimensional approach.^49^ Combining human capital investment (maternal education) with structural assistance (such as economic empowerment and sanitation infrastructure) is likely to yield greater health benefits than addressing each sector separately.

 Furthermore, the consistently low availability of hygienic facilities across all socioeconomic groups suggests that behavioral and social restrictions persist, regardless of financial status, undermining the notion that infrastructure alone is sufficient. Increasing evidence supports multidimensional WASH strategies that combine infrastructure with behaviour change and capacity-building to deliver long-term health outcomes.^50^ Therefore, improving mothers’ educational capacity, combined with measures to enhance economic conditions and WASH infrastructure, appears to be critical to achieving equitable child health outcomes.

 These findings suggest that WASH policies must move beyond coverage metrics and embrace capability-sensitive equity-oriented approaches. Key recommendations include: (1) targeted WASH subsidies for low-income households, (2) integration of maternal health education into sanitation programs, (3) micro-infrastructure investments in underserved regions, (4) national hygiene campaigns that are culturally responsive, and (5) policies aligned with SDG 6 (Clean Water & Sanitation) and SDG 10 (Reduced Inequalities).

 In densely populated urban settlements in Indonesia, especially those with informal or semi-permanent housing clusters, household-level sanitation improvements are often constrained by space, ownership, and affordability issues. A community-based approach is undoubtedly more feasible and impactful. We strongly recommend the development of communal sanitation facilities within densely populated neighborhoods, with support from local governments and community participation. These facilities must meet minimum health and gender-sensitive standards and be complemented with a reliable centralized clean water supply system that does not rely on increasingly contaminated shallow groundwater. Piped surface water or treated rainwater harvesting systems should be introduced for household and communal use wherever feasible. Integrated WASH strategies are key to overcoming infrastructural and socioeconomic barriers to improved sanitation and hygiene in vulnerable communities.^51^ These measures must align with the Community-based Total Sanitation (CBTS) framework and be supported through an intersectoral collaboration between urban planning, public health, and water utilities.

 Despite the advantages of a nationally representative dataset and a multilevel analytical technique, this study has several limitations. First, the cross-sectional design of the study precludes causal correlation. The observed connections between WASH conditions, maternal education, and childhood diarrhea reflect a particular point in time; therefore, temporality and directionality are undefined. Reverse causality and unmeasured confounding cannot be excluded. Second, using mother self-reports to evaluate the incidence of diarrhea during a two-week recall period raises the possibility of recall bias, which could alter the accuracy of prevalence estimations. Although this approach is commonly used in large-scale surveys, misclassification remains possible, such as under- or over-reporting, which can introduce measurement error into the analysis. Third, several hygiene-related indicators were based on proxy factors (such as the availability of handwashing facilities) rather than direct behavioral observations, which may not accurately represent hygiene behaviors. Fourth, while the multilevel model includes geography as a contextual component, it does not account for meso-level determinants, such as local governance quality or subnational variance in WASH program coverage, both of which may impact health outcomes.

 Further longitudinal and mixed-methods research is recommended to investigate causal pathways and behavioral mechanisms more thoroughly. Exploring how hygiene practices and water use mediate these relationships could provide a more comprehensive understanding of the risk of developing diarrhea. Sensitivity analyses confirmed the robustness of key biological determinants (e.g., child’s age, gender, and maternal age) and highlighted the amplified effects of maternal literacy and sanitation access in certain subpopulations. These findings underscore the necessity of targeted context-specific interventions.

**Highlights**

- Toddlers aged 12-23 months face heightened vulnerability to diarrhea.
- Low birth weight compounds early-life health risks.
- Maternal education gaps amplify sanitation-related risks.
- Poverty undermines health knowledge benefits for educated mothers.
- There are persistent regional disparities in childhood diarrhea burden.

## Conclusion

 This study strengthens the evidence that childhood diarrhea in Indonesia is shaped by intersecting biological, socioeconomic, and regional factors, with maternal education and access to WASH as key mediators. The findings challenge infrastructure-centric public health models by demonstrating that infrastructure must be matched with the necessary capabilities, knowledge, and contextual support.

 Future WASH and child health policies should adopt an equity and empowerment lens, targeting intersectional vulnerabilities through integrated context-sensitive strategies that promote empowerment and equity. Only by moving beyond access to effective and inclusive use can Indonesia and other LMICs achieve the sustainable health gains envisioned in global development agendas.

## Acknowledgements

 We appreciate the research team’s hard work for their meticulous efforts in gathering literature, conducting data analysis, and preparing the manuscript. We are also grateful to all collaborators whose substantial contributions enriched the quality of our findings.

## Competing Interests

 The authors declare no conflict of interests.

## Ethical Approval

 This study was ethically approved by Institutional Review Board (IRB) of ICF International (FWA00000845) in compliance with the United States Department of Health and Human Services’ regulations on the “Protection of Human Subjects” (45 CFR 46).

## Funding

 This study received no external funding.

## Supplementary Files

Supplementary file 1 contains Figure S1 and Table S1.
