# Supplementary material for: Beyond Access to Sanitation Services: How Maternal Education Moderates Childhood Diarrhea Risk in Indonesia’s Multilevel Context
Source: J Res Health Sci. 2025 Oct 18;26(1):e00670. doi: 10.34172/jrhs.11228 (PMC12681059; doi:10.34172/jrhs.11228)
Supplement: Supplementary file 1 — contains Figure S1 and Table S1. [file jrhs-26-e00670-s001.pdf]

JRHS
Journal of Research in Health Sciences
doi:10.34172/jrhs.11228
JRHS 2026; 26(1):e00670-S1

Supplementary file 1

Table S1: Sensitivity Analysis of Multilevel Logistic Regression Model for Diarrhea Risk, Using 30% Random Subset.

**Variabels (n=4,990)**	**Model 4 (with interaction)**	
	**OR (95%CI)**	***P*-value**
Child's age (months)		
48–59	Ref.	
36–47	1.15 (0.77, 1.71)	0.494
24–35	2.03 (1.36, 3.04)	0.001
12–23	3.43 (2.14, 5.52)	0.000
0–11	1.42 (0.95, 2.12)	0.085
Child's gender		
Female	Ref.	
Male	1.39 (1.09, 1.79)	0.008
Child's birth weight (g)		
≥ 2,500	Ref.	
< 2,500	1.42 (0.94, 2.16)	0.097
Mother's age (yr)		
35–49 years	Ref.	
25–34 years	0.95 (0.71, 1.28)	0.758
15–24 years	2.11 (1.42, 2.42)	0.000
Mothers's educational level		
Higher	Ref.	
Secondary	1.08 (0.76, 1.54)	0.656
No formal education/primary	1.38 (0.78, 2.42)	0.267
Household wealth		
Richest	Ref.	
Richer	1.54 (0.98, 2.41)	0.062
Middle	1.18 (0.75, 1.87)	0.477
Poorer	0.90 (0.45, 1.83)	0.775
Poorest	0.90 (0.44, 1.83)	0.773
Drinking water		
Improved	Ref.	
Unimproved	1.03 (0.69, 1.53)	0.874
Toilet facilities		
Improved	Ref.	
Unimproved	1.20 (0.73, 1.98)	0.464
Hygiene facilities		
Improved	Ref.	
Unimproved	0.95 (0.75, 1.21)	0.672
Residencial are		
Urban	Ref.	
Rural	1.00 (0.75, 1.33)	0.979
Region		
Java-Bali	Ref.	
Non-Java-Bali	1.43 (1.05, 1.95)	0.023
Interaction terms		
Unimproved toilet × low-educated mother	1.96 (1.04, 3.68)	0.037
Low-wealth index × educated mother	1.55 (0.81, 2.95)	0.185
AIC	4057.151	-
BIC	4213.516	-

ICC (household)	0.552	-
MOR (household)	6.82	-
ICC (cluster)	0.090	-
MOR (cluster)	2.17	-

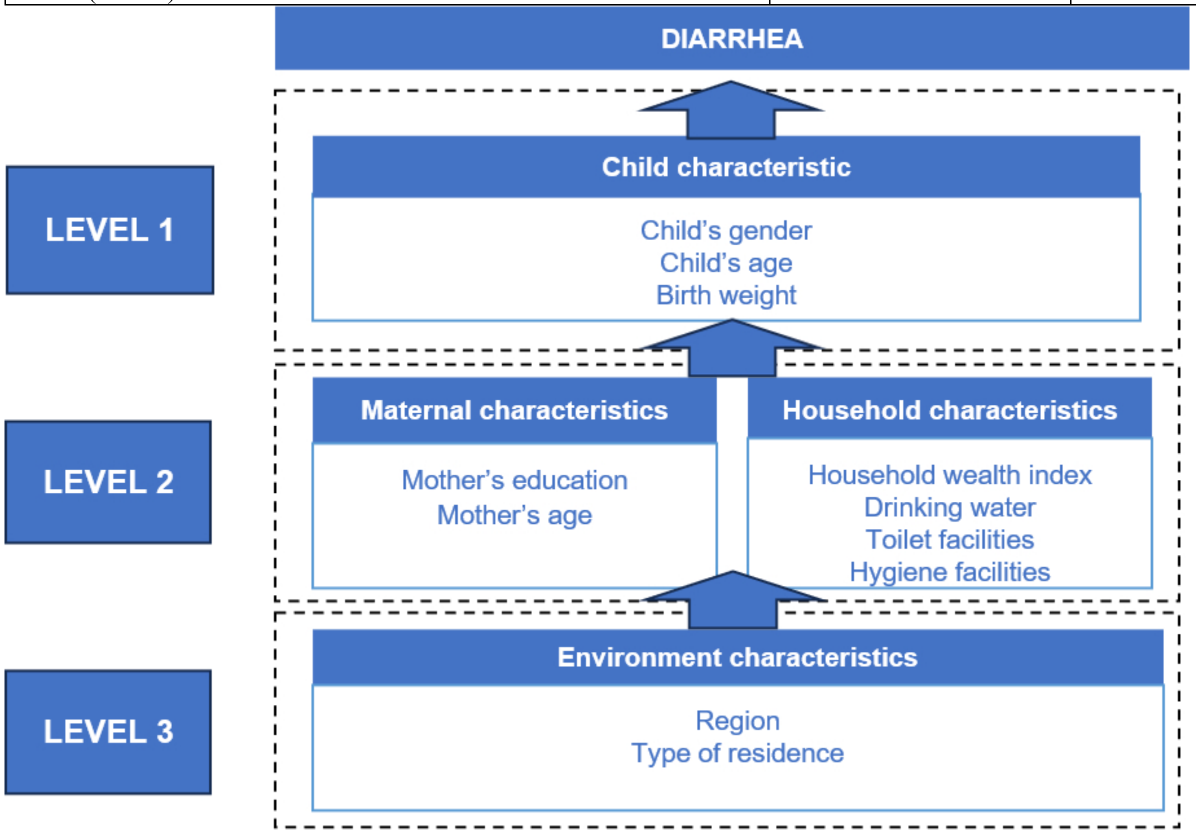

Figure S1: Conceptual framework of multilevel determinants of childhood diarrhea
